# Supplementary material for: Temperature-dependent dual-mode thermal management device with net zero energy for year-round energy saving
Source: Nat Commun. 2022 Aug 19;13:4874. doi: 10.1038/s41467-022-32528-1 (PMC9391366; doi:10.1038/s41467-022-32528-1)
Supplement: Supplementary file 1 — Supplementary Information [file 41467_2022_32528_MOESM1_ESM.pdf]

Temperature-Dependent Dual-Mode Thermal Management Device with Net Zero Energy for Year-Round Energy Saving

Quan Zhang[1], Yiwen Lv[1], Yufeng Wang[1], Shixiong Yu[1], Chenxi Li[2], Rujun Ma[1]*, Yongsheng Chen[2]*

[1]School of Materials Science and Engineering, National Institute for Advanced Materials, Nankai University, Tongyan Road 38, Tianjin, 300350 P. R. China

[2]State Key Laboratory and Institute of Elemento-Organic Chemistry, Centre of Nanoscale Science and Technology and Key Laboratory of Functional Polymer Materials, College of Chemistry, Nankai University, Tianjin, 300071 P. R. China

These authors contributed equally: Quan Zhang, Yiwen Lv

*E-mail: malab@nankai.edu.cn (R. Ma), yschen99@nankai.edu.cn (Y. Chen)

Supplementary Note 1. Numerical modelling analysis of thermal management performance

There is a thermal balance relationship between thermal management device and the ambient as shown in Fig. 1a[1]. The net heat flux of device (P_{net}) is given by

$$P_{net}\left(T_{device},T_{amb}\right)=P_{sun}+P_{atm}\left(T_{amb}\right)-P_{device}\left(T_{device}\right)-P_{parasitic}\left(T_{device},T_{amb}\right) \tag{1}$$

Here, P_{sun} is absorbed solar radiation of the device, $P_{atm}(T_{amb})$ is absorbed thermal radiation of the device from the atmosphere, $P_{device}(T_{device})$ is thermal radiation of the device, and $P_{parasitic}(T_{device},T_{amb})$ is the parasitic heat, namely heat exchange between the device and the ambient by heat conduction and heat convection. Spontaneous thermal radiation of the device is determined by the temperature (T_{device}) and the emissivity of the device ($\varepsilon_{device}(\lambda,\theta)$) simultaneously.

$$P_{device}(T_{device})=A_{device}\int d\Omega\cos\theta\int_0^{\infty}d\lambda I_{BB}\left(T_{device},\lambda\right)\varepsilon_{device}\left(\lambda,\theta\right) \tag{2}$$

A_{device} is the area of the device and $\int d\Omega=2\pi\int_0^{\pi/2}d\theta\sin\theta$ is the angular integral over a hemisphere. $I_{BB}\left(T_{device},\lambda\right)=\frac{2hc^2}{\lambda^5}\frac{1}{e^{\frac{hc}{\lambda k_B T_{device}}}-1}$ is the radiative intensity from a blackbody at temperature (T_{device}), where h is Planck's constant, c is the velocity of light, k_B is the Boltzmann constant, and λ is the wavelength. Radiative heat absorption of the device from the ambient is related to the emissivity of the device and the emissivity of the atmosphere ($\varepsilon_{atm}(\lambda,\theta)$).

$$P_{atm}\left(T_{amb}\right)=A_{device}\int d\Omega\cos\theta\int_0^{\infty}d\lambda I_{BB}\left(T_{amb},\lambda\right)\varepsilon_{device}\left(\lambda,\theta\right)\varepsilon_{atm}\left(\lambda,\theta\right) \tag{3}$$

The angle-resolved emissivity of the atmosphere is $\varepsilon_{atm}\left(\lambda,\theta\right)=1-t_{atm}\left(\lambda\right)$, where $t_{atm}(\lambda)$ is the atmospheric transmissivity in the zenith direction. Absorption from solar radiation ($I_{sun}(\lambda)$) is an important heat source causing the temperature change of the device, which is given by

$$P_{sun}=A_{device}\int_0^{\infty}d\lambda\varepsilon_{device}\left(\lambda,\theta\right)I_{sun}\left(\lambda\right) \tag{4}$$

Non-radiative heat exchange, including heat conduction and heat convection, is linearly

relative to the combined non-radiative heat coefficient (h_c), which represents the collective effect of conductive and convective heat exchange.

$$P_{parasitic}\left(T_{device},T_{amb}\right)=A_{device}h_c\left(T_{device}-T_{amb}\right) \tag{5}$$

Supplementary Note 2. Influence of size of dual-mode device on effectiveness of thermal loading

The size of dual-mode device is indeed an important factor in the effectiveness of thermal loading, switching, and potential applications of the devices. The nature of the issue is the influence of size on the morphological evolution of RC tape-2W SMP film.

Without considering gravity, the radius of curvature of RC tape-2W SMP laminate is length-independent, and depends on the difference of length between RC tape and 2W SMP at different temperatures. It means that the longer RC tape-2W SMP laminate, the higher ratio of active area in the device. The device with large size would have an effectiveness of thermal loading close to the prediction. But in the real world, gravity is not a neglectable factor for the morphological evolution of RC tape-2W SMP laminate, especially if it is very long. In this case, the radius of curvature becomes larger with the increase of gravity. RC tape-2W SMP film can not even completely roll up in extreme conditions. Instead of increasing, the effectiveness of thermal loading of device falls dramatically.

A study on the temperature of the device in heating mode with different sizes is done, thereby visually showing the influence of the size on the heating performance (Supplementary Fig. 21). The radius of curvature is almost the same, when RC tape-2W SMP films are within the limited length (from 10 mm to 40 mm in this experiment). The coiled RC tape-2W SMP films with the same radius of curvature will shade the same area of nano-Cr black Al plate. In other words, the device with the larger size has the higher solar heating power close to the prediction. As one might expect, the temperature rise of the device gradually decreases with the shrinking of active area in the device. The size of the device in next experiments is fixed at 40 mm × 40 mm to achieve an efficient thermal management performance.

Supplementary Note 3. Simulated test of dual-mode device without solar radiation indoors

To reveal the change of infrared emission power of dual-mode device in heating and cooling modes, an indoor experimental setup is designed to monitor temperature and estimate infrared emission power with the assistance of a feed-back controller (Supplementary Fig. 12). The whole setup is sealed by a polyethylene (PE) film in a thermal insulating foam container to minimize the impact from thermal conduction and convection. Two same setups are used in parallel, where one is coated by a dual-mode device and the other is a same-sized aluminum (Al) foil with an infrared emissivity close to zero (Supplementary Fig. 13).

For measurement of infrared emission power, a constant current source is supplied to the Al foil increasing from 0.1 A to 0.24 A with a step of 0.02 A, and the feed-back controller applies an offset Joule heating power on the dual-mode device to minimize the temperature difference between the dual-mode device and the Al foil (Supplementary Fig. 15). The excess of Joule heating power (negative heat flux) is defined as the cooling power of dual-mode device from infrared radiation. As shown in Supplementary Fig. 16a, there is a cooling power of dual-mode device lower than 20 W m^{-2} at lower temperature, which enhances slightly with the temperature increase, when the temperature is lower than 51 °C. Once the temperature is larger than 51 °C, the cooling power rises steeply accompanying the unfold of RC tape-2W SMP film, and it continues to increase with the further increase of the temperature. The inflection temperature of 51 °C corresponds to the transformation of dual-mode device from heating mode to cooling mode. According to the infrared spectral characteristics of dual-mode device, the theoretical cooling powers of dual-mode device in two different modes is predicted (red line is heating mode and blue line is cooling mode). The experimental data agrees well with theoretical prediction in heating mode. It benefits from the negligible contact area between coiled RC tape-2W SMP film and Nano-Cr black coated Al plate, which maximums the effective area of dual-mode device for

thermal management as much as possible. The lack of cooling power in cooling mode is primarily due to the insufficient thermal contact between unfolded RC tape-2W SMP film and Nano-Cr black coated Al plate, which decreases the cooling power of dual-mode device in cooling mode in certain. We also measured the cooling power of dual-mode device at different temperatures during the cooling process (Supplementary Fig. 16b). There is a hysteresis of switching temperature inflection for cooling power between the heating and cooling processes. It is consistent with the hysteresis phenomenon of reversible shape memory of 2W SMP with the temperature (Supplementary Fig. 10).

Meanwhile, we investigated the influence on temperature, when dual-mode device switches from heating mode to cooling mode in this situation (Supplementary Fig. 17). The ambient temperature is ~29 °C and the calculated parasitic heat transfer coefficient (h_c) is 17.5 W m^{-2} K^{-1} (Supplementary Fig. 17a). The temperature of dual-mode device in heating mode is almost the same as that of Al foil. It validates that dual-mode device in heating mode has a very low heat radiative property close to ideal for solar heating. When the temperature is over 50 °C, the dual-mode device gradually switches to cooling mode resulting in the temperature difference between Al foil and dual-mode device. And this difference becomes larger and larger with the increase of Joule heating power ($P_{\text{Joule heating}}$). The temperature difference could disappear again as the dual-mode device gets back into heating mode with the temperature decrease. The whole evolution process is consistent with that of cooling power, including the hysteresis phenomenon. However, these facts show that the ability to radiate heat of dual-mode device exhibits a significant conversion, during the device switches back and forth between two thermal management modes.

Supplementary Note 4. Radiative heat loss and solar absorption

To have an obvious understanding of the impact of outer space and the sun in the nature on solar heating and radiative cooling performance of dual-mode device, we performed an analysis in theory based on the thermal balance relationship (Supplementary Equation (1)). This impact results in a certain difference between the simulated test indoors and the field test outdoors, although a simulated scene can be built indoors with the help of a solar simulator.

In the indoor environment, the device is surrounded by a roof, walls and some other objects, which have a combined infrared emissivity close to 100% in general. Considering this situation, therefore, the overall indoor environment can be considered as a room-temperature (T_{amb}) thermal radiative source. This simplifies Supplementary Equation (3) into $P_{atm}(T_{amb}) = \pi A_{device}\int_0^{\infty} d\lambda I_{BB}(T_{amb},\lambda)\varepsilon_{device}(\lambda)$. In the outdoor environment, the existence of outer space at low temperature makes dual-mode devices have radiative heat loss by reducing the input of infrared radiation from the ambient to the device. Radiative heat absorption of the device from the ambient is only from the atmosphere, while the radiative radiation from out space is negligible due to its extremely cold temperature. In this scene, Supplementary Equation (3) is reducible to $P_{atm}(T_{amb}) = \pi A_{device}\int_0^{\infty} d\lambda I_{BB}(T_{amb},\lambda)\varepsilon_{device}(\lambda)(1 - t_{atm}(\lambda))$, where $t_{atm}(\lambda)$ is the atmospheric transmissivity in the zenith direction.

The input solar power is supplied by a simulated solar source with a spectral distribution of AM 1.5. In the dark (no simulated sunlight), the solar radiation is zero, so the absorption of device from solar radiation (P_{sun}) is also zero. And under the simulated sunlight, $I_{sun}(\lambda)$ is replaced by the spectral distribution of AM 1.5, which is used to calculate the absorption of device from solar radiation (P_{sun}) by Supplementary Equation (4).

Supplementary Fig. 20a shows the predicted heat flux in the dark of dual-mode device without solar radiation in heating and cooling modes indoors and outdoors. From the perspective of dual-mode thermal management, this extra radiative heat loss points

to the importance of reducing the infrared emissivity for solar heating. Meanwhile, an effective way arises for radiative cooling. On the other hand, solar radiation is a considerable energy source able to cause localized heating (Supplementary Fig. 20b). According to the solar radiation with ASTM G173 global solar spectrum, the huge potential on solar-thermal conversion is shown for dual-mode device in heating mode with a high solar absorption. On the contrary, high solar reflection is an essential spectral characteristic of dual-mode device in cooling mode for realizing radiative cooling under solar radiation.

Supplementary Note 5. Effect of incident angle of solar radiation on solar-thermal conversion

In this design, the switch of thermal management modes is realized by morphological evolution of RC tape-2W SMP film. For heating mode, the film rolls to one side to maximize the exposed area of the nano-Cr black Al plate. And for cooling mode, the film spreads out until completely covering the bottom nano-Cr black Al plate. The device in cooling mode is almost isotropic in two-dimensional working plane. By contrast, when the device works in heating mode, the coiled film shields a part of nano-Cr black Al plate away from the solar radiation. This will lower the solar-thermal conversion efficiency of the device per area. Thus, it is very important to carefully select the relative position between the coiled film and the incident sunlight to improve solar-thermal conversion efficiency of the device in heating mode.

To address this issue, we conducted a set of parallel experiments, where four coiled RC tape-2W SMP films are set on the different edges of nano-Cr black Al plate in four individual devices (Supplementary Fig. 22). Because the testing position is in the northern hemisphere (Tianjin: 38.99N, 117.34E), the sun is south of the device for much of the daytime, especially in winter. As expected, the device with the film on the northern edge achieves the maximum temperature rise, while the device with the film on the southern edge has the minimum temperature rise. When the sun rises in the east, the device with the film on the western edge is hotter than that with the film on the eastern edge. And this temperature relationship switches to the opposite when the sun sets in the west. It should be noted that all RC tape-2W SMP films are locked tightly to maintain a coiled state. The reason is that unconstrained film will completely unfold and the device works at cooling mode in the hot summer. Overall, when the coiled RC tape-2W SMP film is set on the edge opposite to the sun, the device in heating mode could achieve the maximum solar-thermal conversion efficiency without negative effect on cooling ability of the device in cooling mode (namely set on the northern edge when in the northern hemisphere and the southern edge when in the southern

hemisphere). Accordingly, all of the data in field test are achieved when RC tape-2W SMP film is set on the northern edge.

Supplementary Note 6. Estimation of energy saving monthly

Based on the thermal balance relationship between thermal management device and the ambient ((1)), the potential of energy saving of dual-mode device in different climatic zones at different seasons is estimated according to monthly weather data for a typical meteorological year, without consideration of heat transfer in diverse and complex thermal management system in practice application scenes. The meteorological data is achieved from the software of Meteonorm version 8. Solar heating power and radiative cooling power of the dual-mode device is equal to the sum of absorbed solar radiation, the absorbed thermal radiation from the ambient, and the spontaneous infrared emission, corresponding to the electromagnetic spectrum in each thermal management mode. We did not consider much about the influence of humidity on the transmissivity of atmospheric window for mid-infrared radiation. It could result in an overestimation of radiative cooling performance and an underestimation of solar heating performance of dual-mode device in a certain way[2]. Even so, this numerical prediction still reveals the great potential of dual-mode device for energy saving, which design provides a real zero-energy thermal management strategy. We analyzed that the energy saving of the dual-mode device in different modes changes with month in one year in detail, taking Tianjin as an example (Fig. 5b and Supplementary Table 2). Meanwhile, the difference in energy saving in different regions is also compared (Supplementary Fig. 25 and Supplementary Fig. 26a). Fig. 5e and Supplementary Fig. 26b visually show the potential of energy saving in different cities representing typical climatic zones on the earth, where thermal management mode of dual-mode device is determined by average ambient temperature (Supplementary Table 3 and Supplementary Table 4). The dividing temperature is ~17 °C, approximately equal to the average temperature of Beijing in spring or autumn.

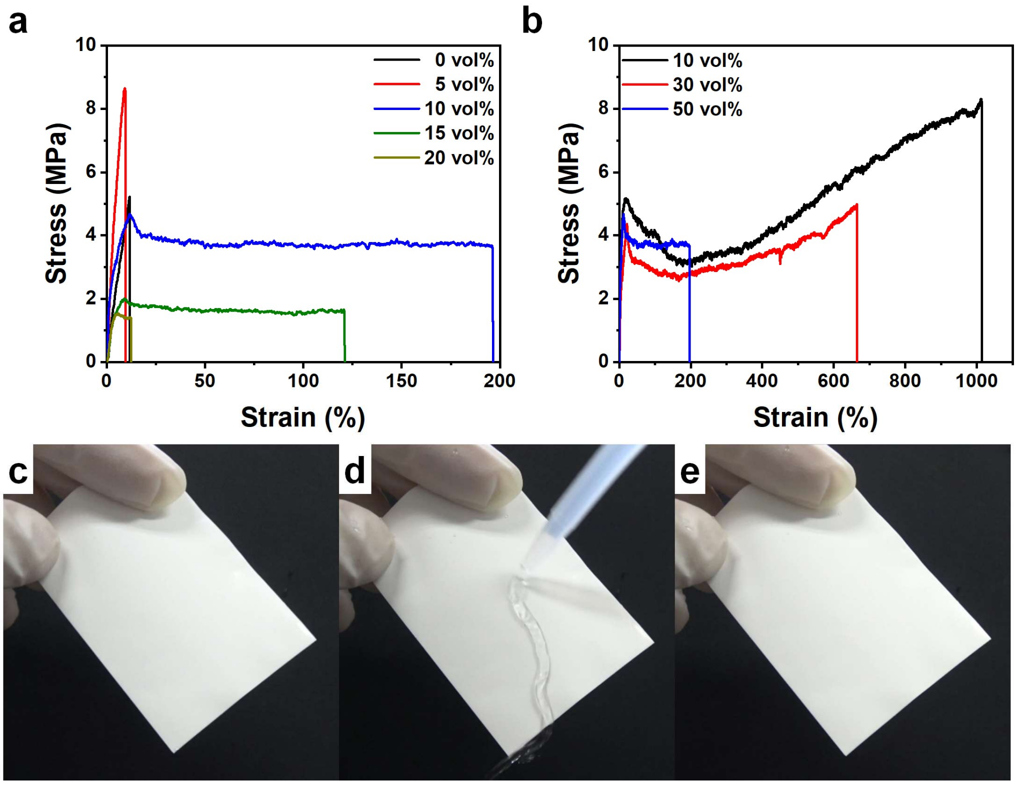

Supplementary Fig. 1 | Mechanical properties and hydrophobicity of DOP-modified PMP encapsulating TiO_2 NPs. a, Stress-strain curves for DOP-modified PMP/TiO_2 NPs composite films with different volume fractions of DOP. The volume fraction of TiO_2 NPs is fixed at 50%. **b**, Stress-strain curves for DOP-modified PMP/TiO_2 NPs composite films with different volume fractions of TiO_2 NPs. The volume ratio between PMP and DOP is fixed at 4:1. **c**-**e**, Photographs of a RC tape showing its appearance (**c**) before, (**d**) during and (**e**) after wetting with deionized water. No changes are observed, indicating that RC tape is hydrophobic.

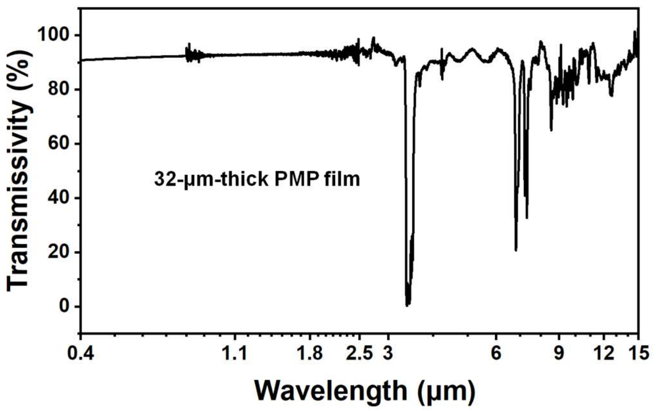

Supplementary Fig. 2 | Measured transmission spectrum of 32-μm-thick PMP film.

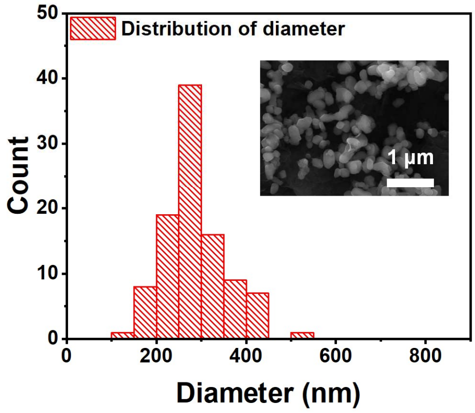

Supplementary Fig. 3 | Size distribution of TiO_2 NPs. The diameter of TiO_2 NPs centers at 200 nm. The inset is a SEM image of TiO_2 NPs.

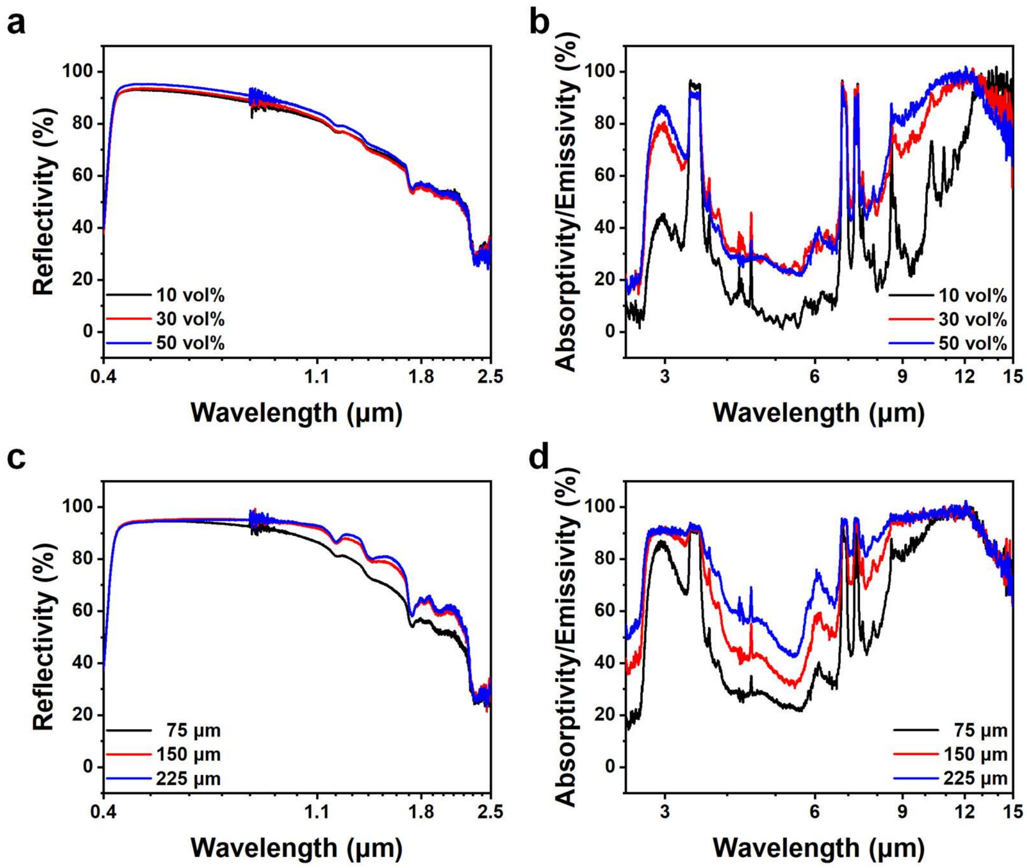

Supplementary Fig. 4 | Spectral characteristics of RC tape. a,**b**, Influence of TiO_2 NPs content on (**a**) reflectivity for solar radiation and (**b**) infrared absorptivity/emissivity. The thickness of RC tape is 75 μm. **c**,**d**, For the same content of TiO_2 NPs (volume fraction of 50%), there is a certain increase in (**c**) solar reflection and (**d**) infrared absorption/emission with the increasing film thickness. The 225-μm-thick DOP-modified PMP encapsulating TiO_2 NPs of 50% volume fraction shows strong solar reflection (weighted average of larger than 90%) and high mid-infrared emission (weighted average of larger than 96%). Even so, 75-μm-thick RC tape with TiO_2 NPs of 30% volume fraction is selected to fabricate dual-mode device to ensure the coiled shape of laminate at the cost of reducing a little solar reflection. The reason is that too thick RC tape will limit its lateral bending due to large moment of inertia, further breaking the coiled shape of RC tape-2W SMP laminate at low temperature.

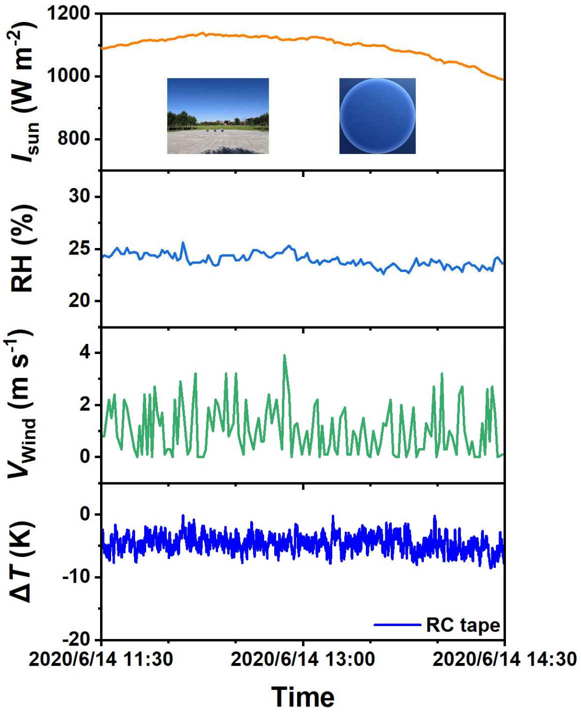

Supplementary Fig. 5 | Time-resolved curves of meteorological data, including intensity of solar radiation (I_{sun}, orange line), humidity (RH, light-blue line) and wind speed (V_{wind}, light-green line), and temperature difference (blue line, $\Delta T = T_{RC\ tape} - T_{amb}$) between RC tape ($T_{RC\ tape}$) and the ambient ($T_{amb}$). An average temperature drop of ~5 K is achieved on a clear sunny day in summer, when RC tape exposes to the sky without any convection shield in a large open square (inset images). Sub-ambient cooling is a necessary performance for radiative cooling materials to save energy at high temperature.

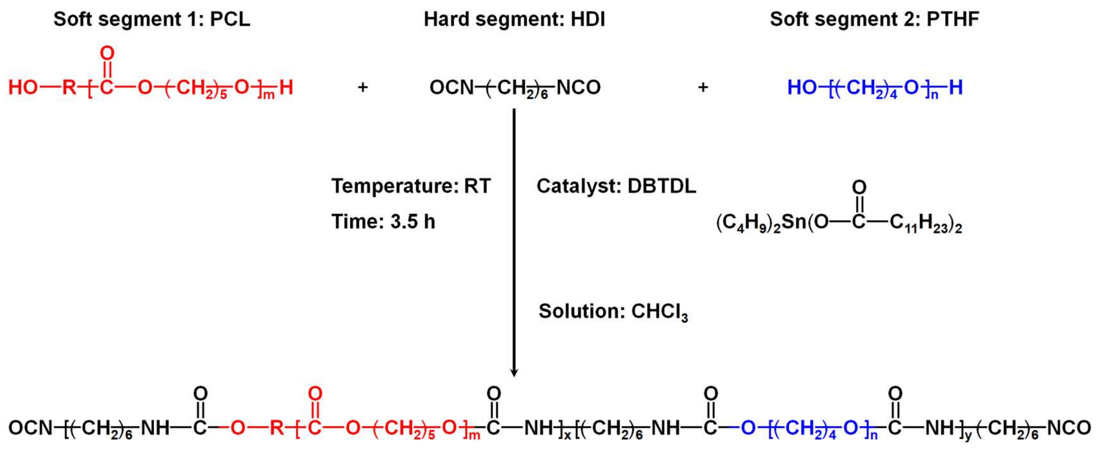

Supplementary Fig. 6 | Synthesis route to 2W SMP.

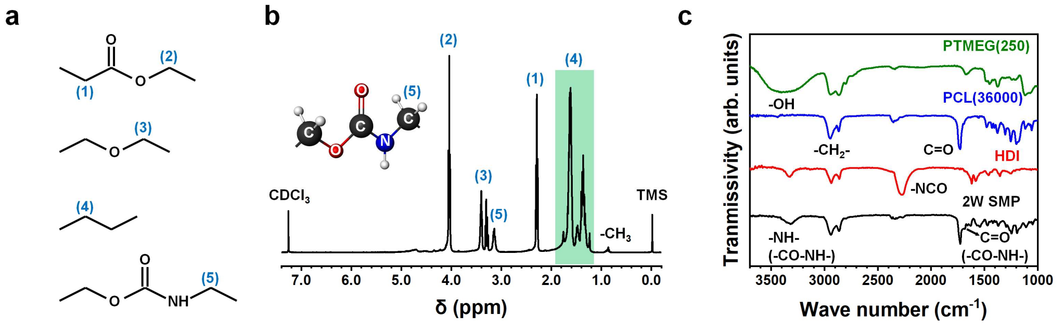

Supplementary Fig. 7 | Characterization of as-prepared 2W SMP. a, Characteristic molecular structures in as-prepared 2W SMP (ester group, ether bond). **b**, 1H NMR spectrum of 2W SMP in $CDCl_3$ (including 0.03% TMS). **c**, FTIR spectrums of 2W SMP and three monomers.

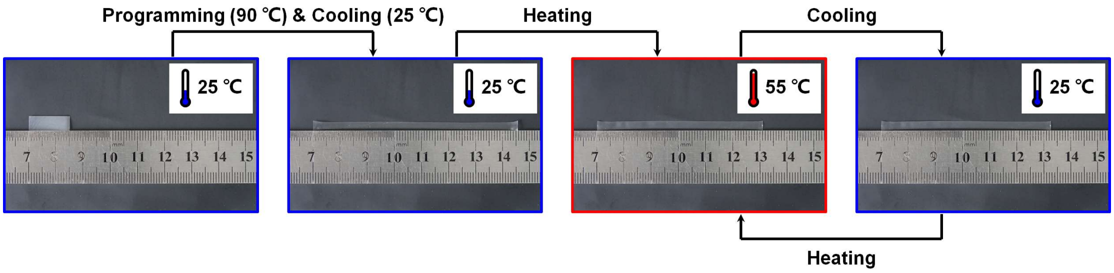

Supplementary Fig. 8 | Optical images of shape programming and reversible actuation of 2W SMP.

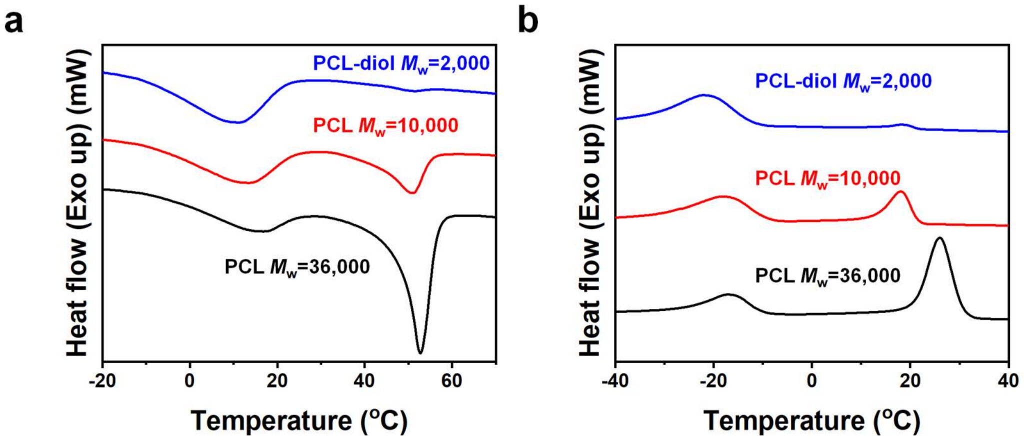

Supplementary Fig. 9 | DSC curves of 2W SMP with molar ratio of three monomers (n_{PTHF}: n_{PCL}: n_{HDI}=9:1:20) in (a) heating and subsequent (b) cooling run at rate of 10 °C min^{-1} after eliminating the thermal history. There are two groups of melting temperature and crystallization temperature for 2W SMP. The lower is from the melting and crystallization of PTHF chain segments and the higher is from PCL segments. For the same molar ratio, two groups of melting temperature and crystallization temperature both shift towards lower temperature with the decrease of molecular weight (M_w) of PCL-like monomer. Meanwhile, the enthalpy of PTHF segments increases and that of PCL segments decreases.

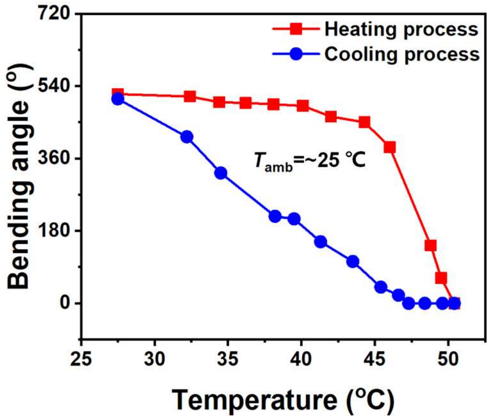

Supplementary Fig. 10 | Reversible bending deformation of RC tape-2W SMP laminate as a function of heating plate temperature. The molecular weight of PCL monomer is 36,000 and the ambient temperature is around 25 °C.

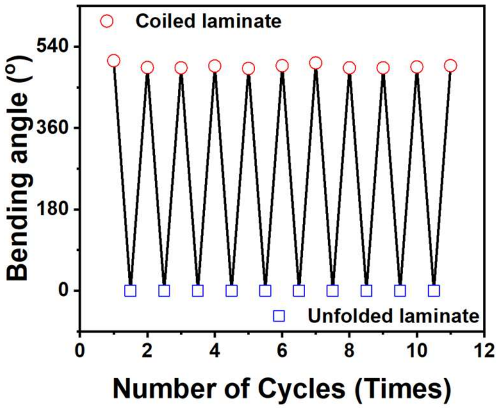

Supplementary Fig. 11 | Reversible bending deformation of a RC tape-2W SMP laminate as a function of the number of cycles with the temperature of heating plate switching between 27 and 51 °C. The molecular weight of PCL monomer is 36,000.

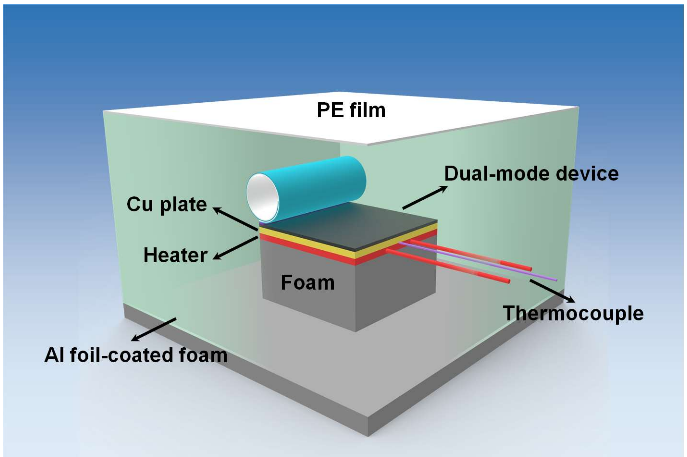

Supplementary Fig. 12 | Experimental setup of dual-mode thermal management performance estimation.

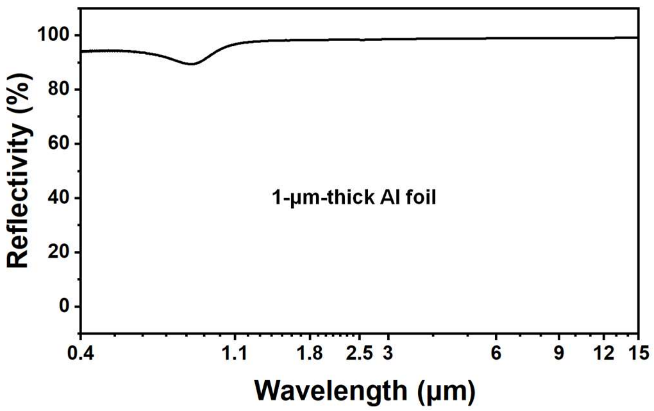

Supplementary Fig. 13 | Reflectance spectrum of 1-μm-thick Al foil simulated by finite difference time domain (FDTD) method.

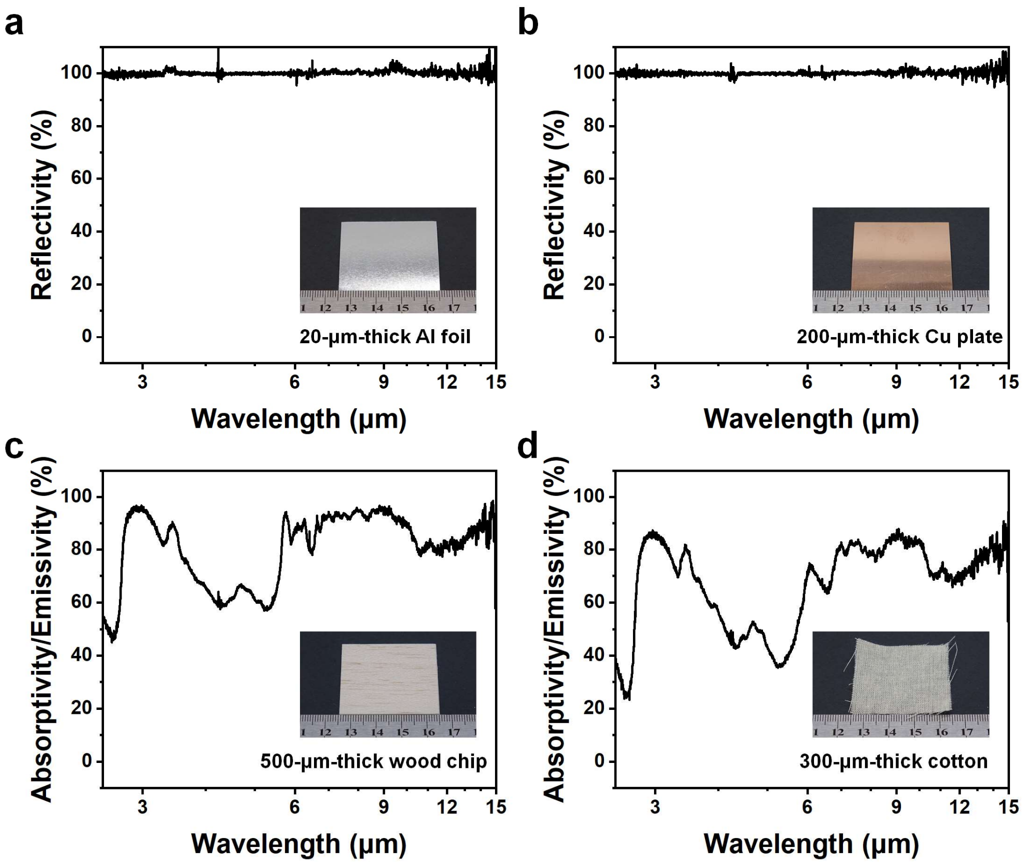

Supplementary Fig. 14 | Spectral characteristics of several reference materials. a,**b**, Reflectance spectra of (**a**) 20-μm-thick Al foil and (**b**) 200-μm-thick Cu plate. **c**,**d**, Absorption/emission spectra of (**c**) 500-μm-thick wood chip and (**d**) 300-μm-thick cotton. The insets are the corresponding optical images.

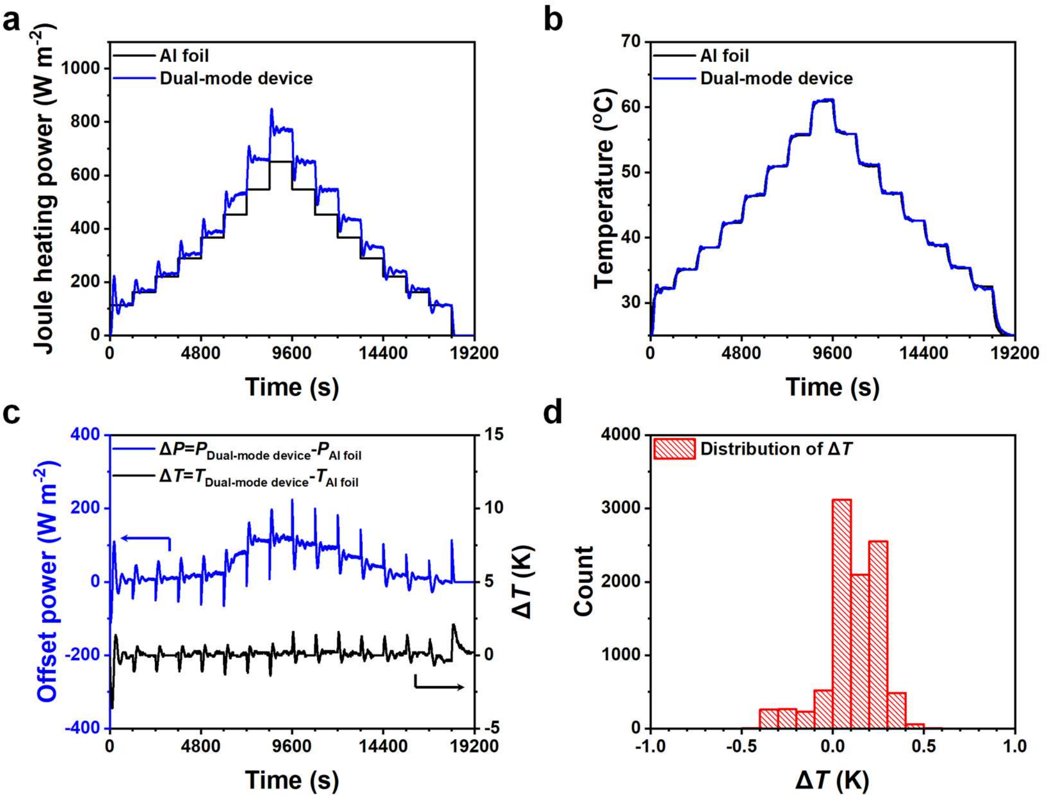

Supplementary Fig. 15 | Time-resolved (a) Joule heating power and (b) corresponding temperature curves of dual-mode device (blue line) and Al foil (black line). c, Time-resolved differences of Joule heating power (ΔP=$P_{\text{Dual-mode device}}$-$P_{\text{Al foil}}$, blue line) and temperature (ΔT=$T_{\text{Dual-mode device}}$-$T_{\text{Al foil}}$, black line) between the dual-mode device and Al foil. **d**, Histogram of temperature difference between dual-mode device and Al foil at the last 300 s in every step of Joule heating power applied on Al foil.

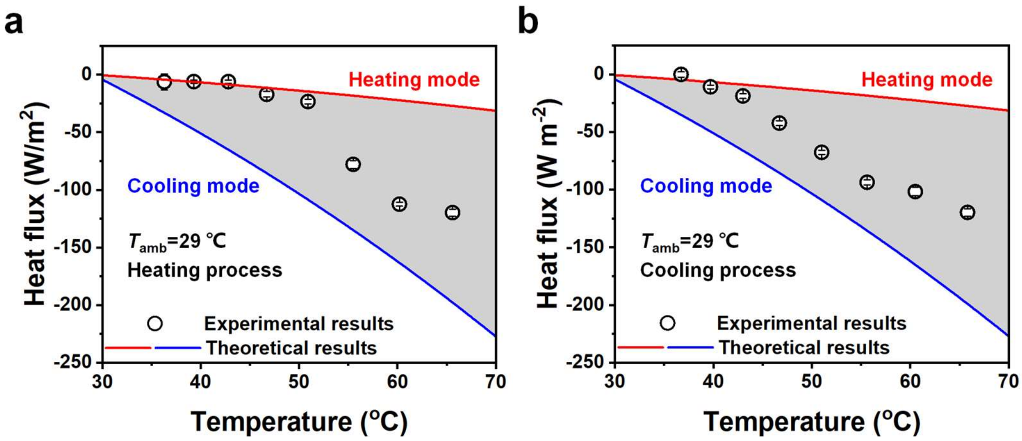

Supplementary Fig. 16 | Change in cooling power of dual-mode device switching between heating (red line) and cooling (blue line) modes indoors. a, Heating process. **b**, Cooling process. The gray area represents the enhancement of thermal radiation from the change in infrared characteristics of dual-mode device switching from heating mode to cooling mode. The ambient temperature fluctuates around 29 ℃ slightly. The error bars represent the standard deviation of data in the last 600 s within each period.

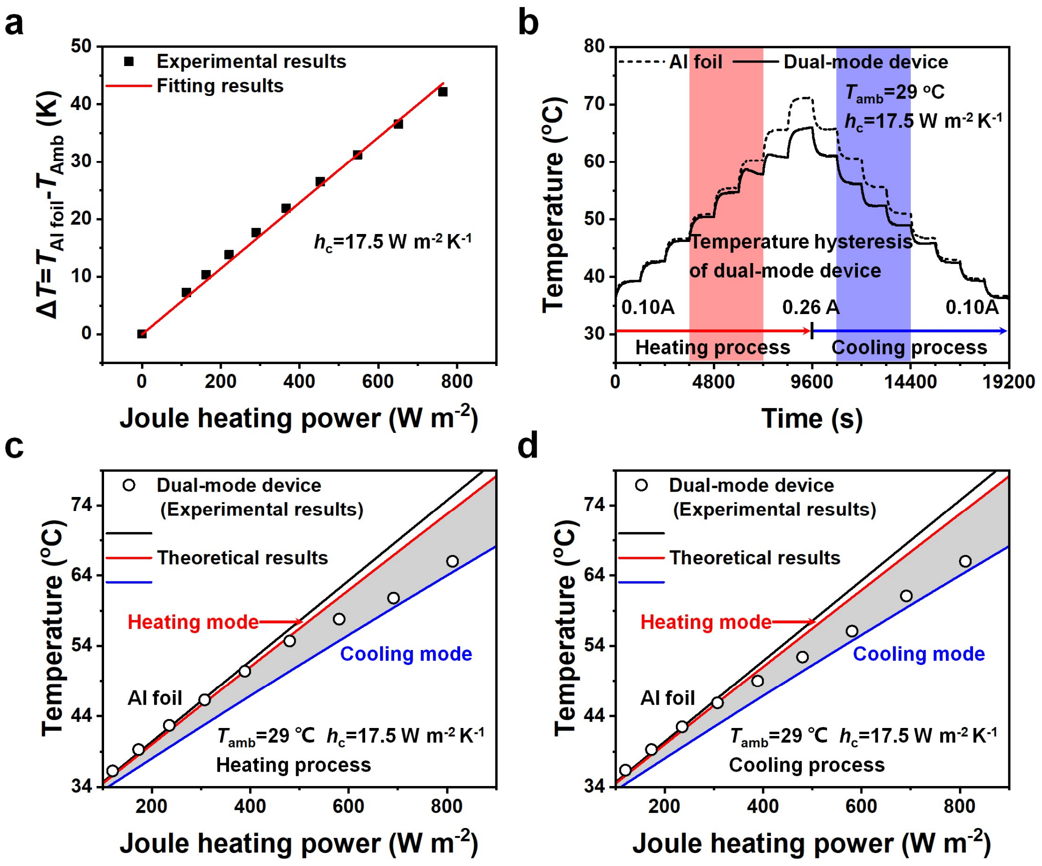

Supplementary Fig. 17 | Change in cooling effect of dual-mode device switching between thermal management modes indoors. a, Temperature difference between Al foil and the ambient (ΔT=$T_{\mathrm{Al\ foil}}$-T_{amb}) as a function of Joule heating power. The reciprocal of the slope is the heat transfer coefficient (h_c) for testing apparatus (Supplementary Fig. 12) in the room, equal to 17.5 W m^{-2} K^{-1}. **b**, Time-resolved temperature curves of dual-mode device (line) and Al foil (dash line), when the applied current increases from 0.1 A to 0.26 A and decreases to 0.1 A again with the step of 0.02 A. **c**,**d**, Temperature of dual-mode device applied with different Joule heating power during (**c**) heating process and (**d**) cooling process. Three solid lines represent the theoretical temperature of Al foil (black line) and dual-mode device in heating (red line) and cooling (blue line) modes, respectively. At lower temperature, measured temperature (black circle) is almost consistent with the theoretical data of dual-mode device in heating mode. It gradually goes near to the theoretical data of dual-mode device in cooling mode, as the Joule heating power increases. The ambient temperature

fluctuates around 29 ℃ slightly.

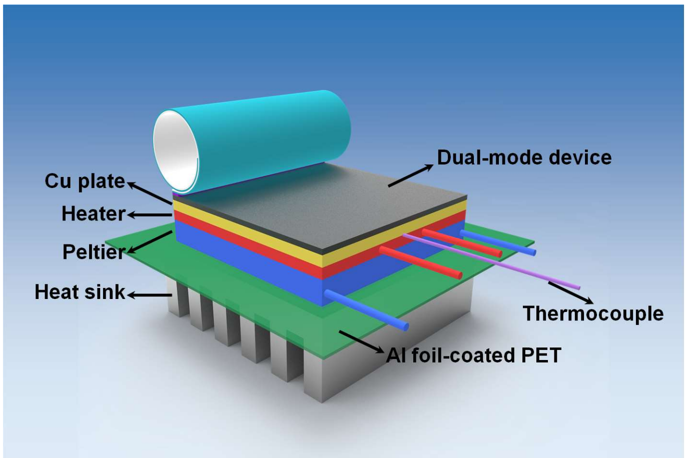

Supplementary Fig. 18 | Schematic of dual-mode heat flux measurement apparatus.

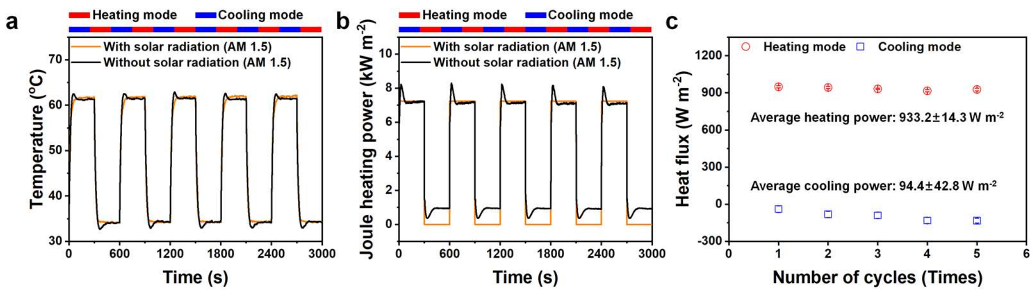

Supplementary Fig. 19 | Indoor thermal management performance of the dual-mode device using AM1.5 solar simulator. a,**b**, Real-time (**a**) temperature of dual-mode device and corresponding (**b**) Joule heating power for 5 heating-cooling cycles. Orange lines are measured under simulated solar radiation (AM 1.5), and black lines are measured in the dark. **c**, Average solar heating power (positive heat flux, red circle ring) and radiative cooling power (negative heat flux, blue square ring) for 5 heating-cooling cycles with the assistance of solar simulator (AM 1.5) indoors. The error bars represent the standard deviation of data in the last 120 s within each period.

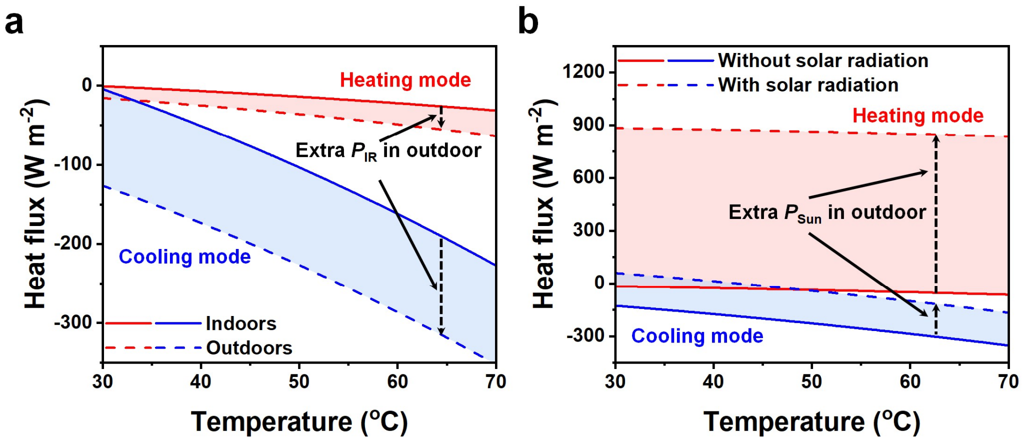

Supplementary Fig. 20 | Influence of outer space and the sun on the thermal management performance of dual-mode device in different modes. The ambient temperature (T_{amb}) is fixed as 29 °C, and the combined non-radiative heat coefficient (h_c) is assumed as 0. **a**, Predicted heat flux in the dark of dual-mode device without solar radiation in heating (red) and cooling (blue) modes indoors (line) and outdoors (dash line). The light-red area and light-blue area represent the extra radiative thermal loss (P_{IR}) of dual-mode device in heating and cooling modes outdoors compared with that indoors. **b**, Predicted heat flux of dual-mode device with (dash line) and without (line) solar radiation (ASTM G173) in heating (red) and cooling (blue) modes outdoors. The light-red area and light-blue area represent the extra solar absorption (P_{sun}) of dual-mode device works in heating (red) and cooling (blue) modes.

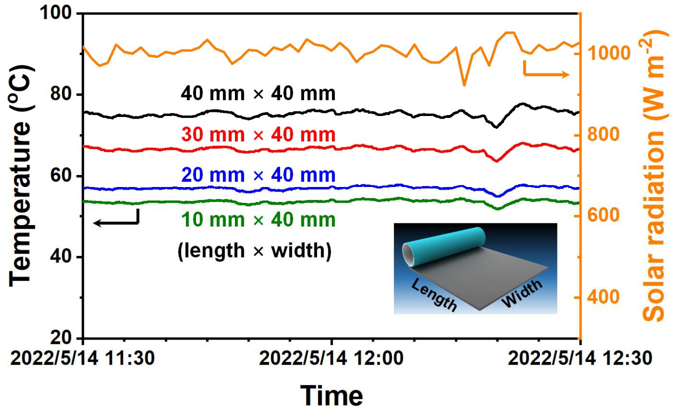

Supplementary Fig. 21 | Influence of size of dual-mode device on effectiveness of thermal loading. Time-resolved temperature curves of dual-mode device in heating mode with different sizes, along with the corresponding solar radiation. It should be noted that all RC tape-2W SMP films are locked tightly to maintain a coiled state. The reason is that unconstrained film will completely unfold and the device works at cooling mode in the hot summer. The coiled RC tape-2W SMP film will shield a part of nano-Cr black Al plate, when the device works in heating mode. For a smaller device, especially the shorten of the length along the unfolding direction of the laminate, the reduction of the active zone in nano-Cr black Al plate decreases the total heating power per area of the device.

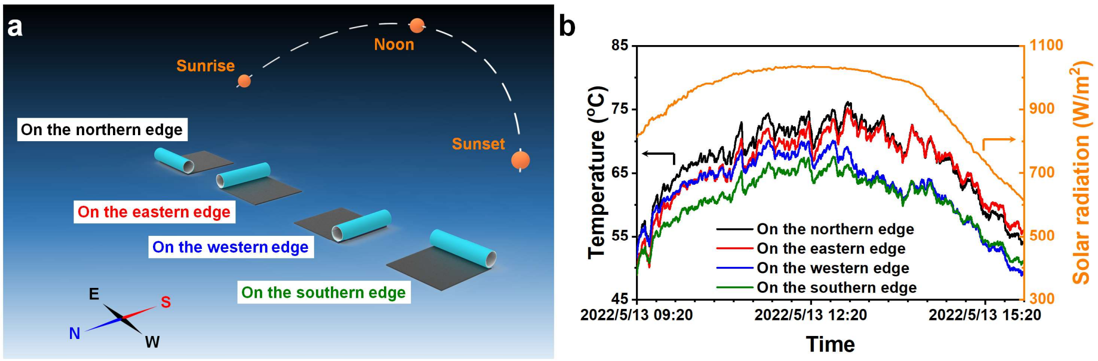

Supplementary Fig. 22 | Effect of incident angle of solar radiation on solar-thermal conversion. **a**, Schematic illustration showing four coiled RC tape-2W SMP films separately set on the different edges of nano-Cr black Al plate in four individual dual-mode devices. Because the testing position is around latitude 39 degrees north, the sun is often south of the device, especially at noon. **b**, Time-resolved curves of intensity of solar radiation (orange line) and temperature of dual-mode devices in heating mode. The coiled RC tape-2W SMP films are set on the different edges of nano-Cr black Al plate in four individual devices: on the northern edge (black line), on the eastern edge (red line), on the western edge (blue line), and on the southern edge (green line).

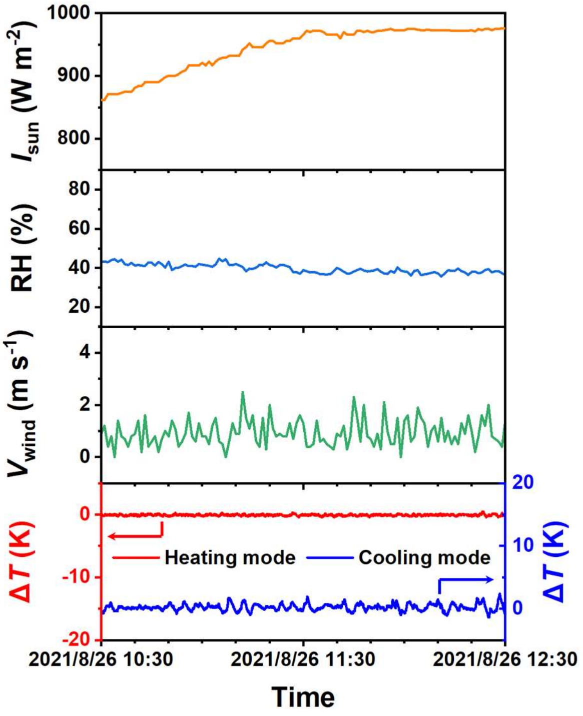

Supplementary Fig. 23 | Time-resolved curves of meteorological data, including intensity of solar radiation (I_{sun}, orange line), humidity (RH, light-blue line) and wind speed (V_{wind}, light-green line), and temperature difference (ΔT) between dual-mode device in heating (red line) and cooling (blue line) mode and Al foil, corresponding to Fig. 5a.

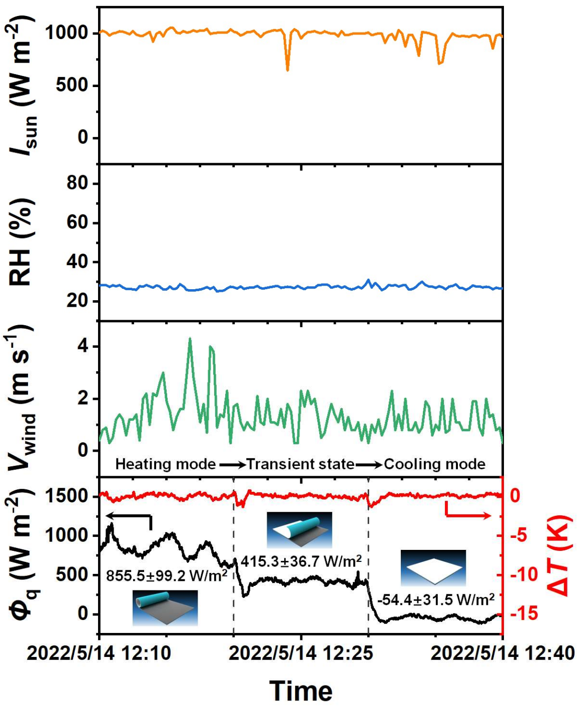

Supplementary Fig. 24 | Time-resolved curves of heat flux (Φ_q) (black line) and temperature difference between dual-mode device and Al foil (ΔT=$T_{\text{dual-mode device}}$-$T_{\text{Al foil}}$) (red line). The device evolves from heating mode to intermediate state, then to cooling mode. The corresponding meteorological data, including intensity of solar radiation (orange line), humidity (light-blue line) and wind speed (light-green line), are also listed.

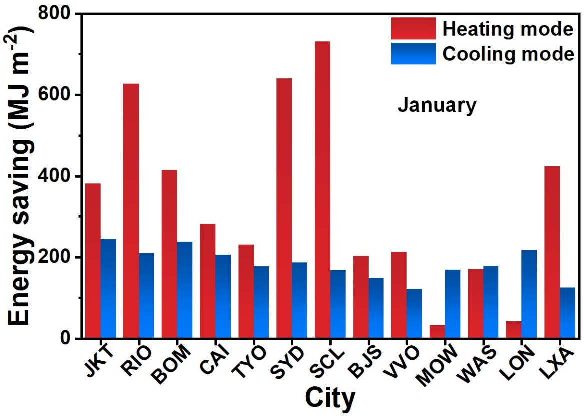

Supplementary Fig. 25 | Modeled all energy saving of dual-mode device in different modes in some cities representing typical terrestrial climatic zones in January. (JKT: Jakarta, RIO: Rio de Janeiro, BOM: Mumbai, CAI: Cairo, TYO: Tokyo, SYD: Sydney, SCL: Santiago de Chile, BJS: Beijing, VVO: Vladivostok, MOW: Moscow, WAS: Washington D.C., LON: London, LXA: Lhasa).

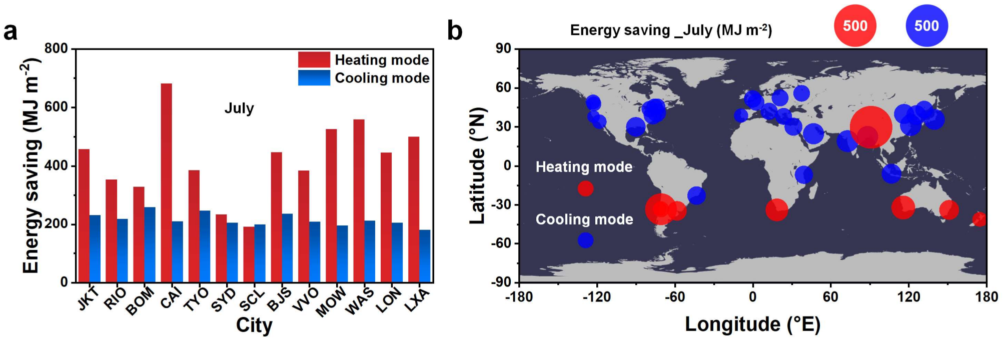

Supplementary Fig. 26 | Modeled energy saving of dual-mode device in some cities representing typical terrestrial climatic zones in July. a, Both heating and cooling modes. (JKT: Jakarta, RIO: Rio de Janeiro, BOM: Mumbai, CAI: Cairo, TYO: Tokyo, SYD: Sydney, SCL: Santiago de Chile, BJS: Beijing, VVO: Vladivostok, MOW: Moscow, WAS: Washington D.C., LON: London, LXA: Lhasa). **b**, Modeled energy-saving (radius of circle) map. The critical temperature for dividing heating (red circle) and cooling (blue circle) modes is assumed as 17 °C, which is approximately equal to the average temperature of Beijing in spring and autumn.

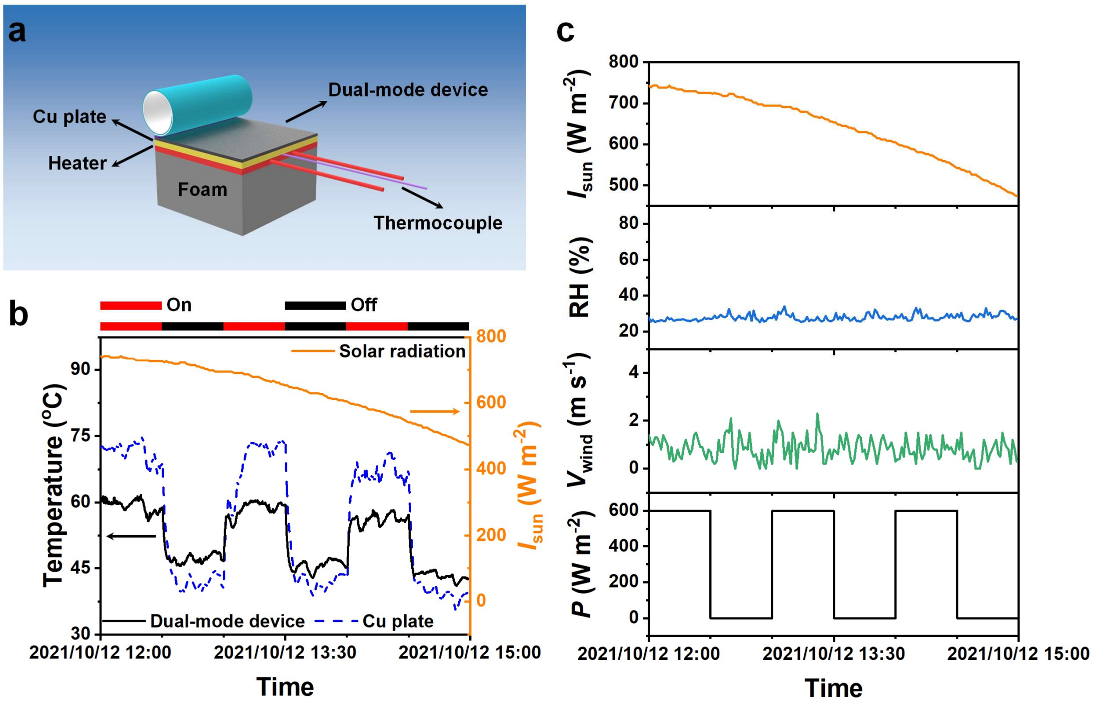

Supplementary Fig. 27 | Demonstration of temperature control by dual-mode device in a clear daytime. a, Schematic of temperature measurement setup for demonstration of thermal management. **b**, Time-resolved temperature curves of dual-mode device (black line) and 200-μm-thick Cu plate (blue dash line), along with corresponding solar radiation (I_{sun}, orange line). As Joule heating power is repeat to be on-off, the dual-mode device switches between cooling mode and heating mode by perceiving temperature. The real-time temperature difference between them is shown in Fig. 5f. **c**, Time-resolved curves of meteorological data, including intensity of solar radiation (I_{sun}, orange line), humidity (RH, light-blue line) and wind speed (V_{wind}, light-green line), and Joule heating power (P, black line).

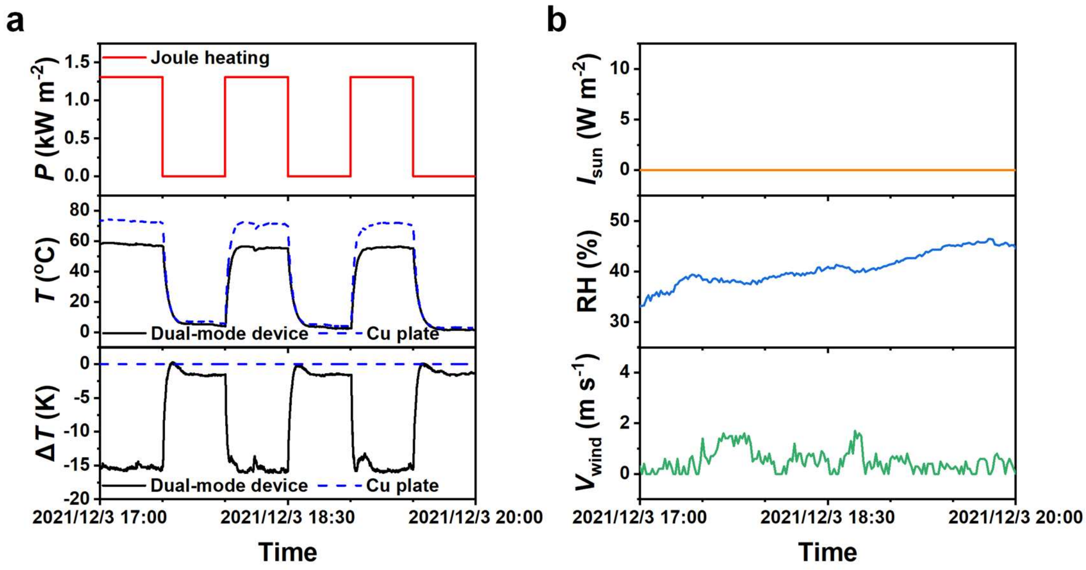

Supplementary Fig. 28 | Demonstration of temperature control by dual-mode device in a clear nighttime. a, Real-time temperature (*T*) and corresponding temperature difference ($\Delta T = T_{\mathrm{sample}} - T_{\mathrm{Cu\ plate}}$) of dual-mode device (black line) compared with 200-μm-thick Cu plate (blue dash line). As Joule heating power (*P*, red line) is repeated to be on-off, the dual-mode device switches between cooling mode and heating mode by perceiving temperature. **b**, Corresponding time-resolved curves of meteorological data, including intensity of solar radiation (I_{sun}, orange line), humidity (RH, light-blue line) and wind speed (V_{wind}, light-green line).

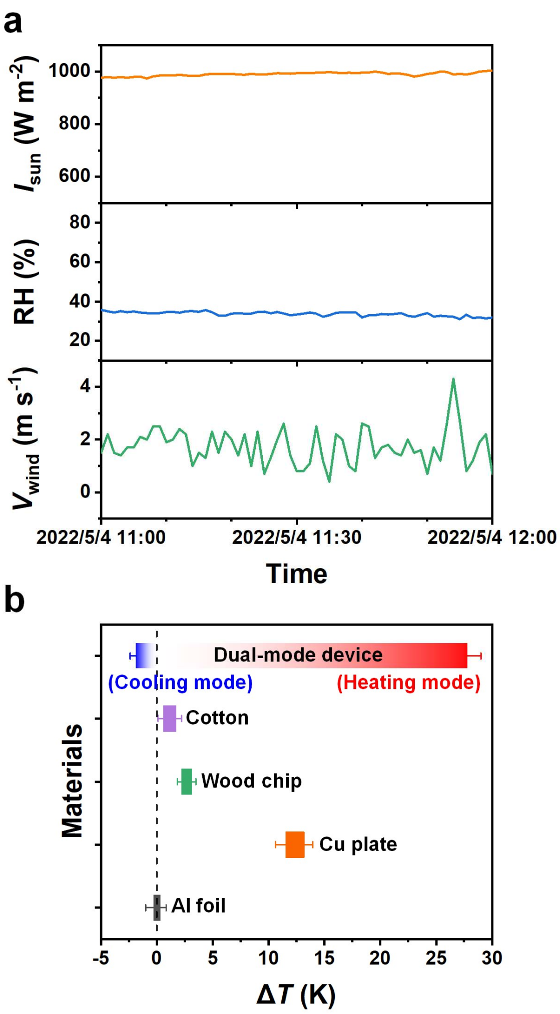

Supplementary Fig. 29 | Expansion of temperature change zone by dual-mode device. a, Time-resolved curves of meteorological data, including intensity of solar radiation (I_{sun}, orange line), humidity (RH, light-blue line) and wind speed (V_{wind}, light-green line). **b**, Temperature change zone of dual-mode device and four common materials with fixed spectral characteristics (corresponding to Supplementary Fig. 14). The average temperature of Al foil is used as reference to calculate temperature change ($\Delta T = T_{sample} - T_{Al\ foil}$). Al foil, gray bar; Cu plate, orange bar; wood chip, green bar; cotton, purple bar; dual-mode device, color-graduated bar (heating mode is red boundary and cooling mode is blue boundary). The error bars represent the standard deviation of data in the last 1200 s within each period.

Supplementary Table 1 | Feature comparison of the dual-mode thermal management devices between in this work and reported in the literature. *Estimated from electromagnetic spectrums.

Device structure	Solar heating/ Radiative cooling	Spectrum for solar heating	Spectrum for radiative cooling	Energy consumption for switching
Dynamic gating[3]	--- / ---	---	---	Zero energy (humidity responding)
Solar absorber on radiative cooler[4]	Yes / Yes	α=80% (0.3-1.8 μm)*<br>ε=10% (8-13 μm)*	α=50% (0.3-1.8 μm)*<br>ε=80% (8-13 μm)*	Unable to switch
Porous P(VDF-HFP)[5]	--- / ---	T=94% (0.4-2.5 μm)<br>T=0.3% (8-13 μm)	T=20% (0.4-2.5 μm)<br>T=64% (8-13 μm)	Mechanical energy
Porous PDMS on CB NPs (bilayer)[6]	Yes / Yes	α=~95% (0.2-2.5 μm)<br>ε=~94% (5-20 μm)	R=~93% (0.2-2.5 μm)<br>ε=~94% (5-20 μm)	Mechanical energy
Integrated structure[7]	Yes / Yes	α=93.4% (0.3-2 μm)<br>ε=14.2% (4-18 μm)	R=97.3% (0.3-2 μm)<br>ε=94.1% (8-13 μm)	Electrical energy
Hybrid structure[8]	Yes / Yes	α=~92% (0.5-2 μm)<br>ε=~10 (2-16 μm)	R=~90% (0.5-2 μm)*<br>ε=~100% (8-13 μm)*	Unable to switch
Janus textile[9]	Yes / Yes	α=>80% (0.3-2.5 μm)<br>ε=~16% (6-20 μm)	R=~91% (0.3-2.5 μm)<br>ε=~87% (2.5-20 μm)	Mechanical energy
Core-Shell Microsphere Structures[10]	--- / ---	---<br>ε=18% (3-14 μm)	---<br>ε=68% (3-14 μm)	Zero energy (temperature responding)
Janus film[11]	Yes / Yes	α=87% (0.38-2.5 μm)<br>α=5% (6-15 μm)	α=4% (0.38-2.5 μm)<br>α=66% (6-15 μm)	Mechanical energy
Phase-changing copolymer[12]	--- / ---	T=87.0% (0.3-2.5μm)<br>---	T=24.3% (0.3-2.5μm)<br>---	Zero energy (temperature responding)
W-doped VO_2 thermochromic window[13]	--- / ---	T=30.0% (0.3-2.5μm)<br>ε=21.0% (8-13 μm)	T=30.0% (0.3-2.5μm)<br>ε=61.0% (8-13 μm)	Zero energy (temperature responding)
W-doped VO_2 thermochromic coating[14]	--- / ---	α=25.0% (0.3-2.5μm)<br>ε=20.0% (8-13 μm)	α=25.0% (0.3-2.5μm)<br>ε=90.0% (8-13 μm)	Zero energy (temperature responding)
Dual-mode device (this work)	Yes / Yes	α=~91% (0.4-2.5 μm)<br>ε=~8% (2.5-15 μm)	R=~85% (0.4-2.5 μm)<br>ε=~97% (2.5-15 μm)	Zero energy (temperature responding)

Supplementary Table 2 | Meteorological data and estimated energy saving potential of Tianjin in different months in one typical year.

Month	Average temperature (°C)	Global solar radiation (MJ m^{-2})	Energy saving (MJ m^{-2}) (Heating mode/Cooling mode)
January	-3	223.2	181.2/152.6
February	0.4	288	241.1/137.3
March	8	406.8	342.9/166.5
April	15.4	511.2	434.2/173.7
May	21.9	619.2	527.0/195.5
June	25.5	568.8	480.0/211.0
July	27.6	543.6	454.4/239.3
August	26.4	486	403.1/238.3
September	21.8	428.4	355.2/215.2
October	14.4	334.8	273.9/205.0
November	5.6	223.2	187.8/178.1
December	-1.1	187.2	148.4/165.1

Supplementary Table 3 | Meteorological data and estimated monthly energy saving potential of different cities in January. The critical temperature for determining the working mode of dual-mode device is assumed as 17 °C, approximately equal to the average temperature in Beijing in spring or autumn.

Cities	Longitude (°E)/ Latitude (°N)	Climatic zone	Average temperature (°C)	Global solar radiation (MJ m^{-2})	Energy saving (MJ m^{-2}) (Heating mode/ Cooling mode)
Jakarta	106.8/-6.1	Tropical rainy climate	26.3	460.8	381.9/243.9
Rio de Janeiro	-43.3/-22.9	Tropical savanna climate	27.4	730.8	626.8/209.0
DaresSalaam	39.3/-6.9	Tropical savanna climate	28.2	705.6	603.3/216.8
Mumbai	72.9/19	Tropical monsoon climate	26.1	496.8	414.8/237.5
Kolkata	88.3/22.5	Tropical monsoon climate	18.7	446.4	374.0/209.3
Cairo	31.3/30.1	Tropical desert climate	14.5	342.0	281.6/205.9
Riyadh	46.8/24.7	Tropical desert climate	14.7	532.8	455.1/178.2
Shanghai	121.5/31.2	Subtropical monsoon climate	4	234.0	189.2/178.3
Tokyo	139.8/35.7	Subtropical monsoon climate	5.4	280.8	231.0/176.8
New Orleans	-90.1/30	Subtropical monsoon climate	12.2	298.8	243.6/202.4
Sao Paulo	-70.7/-33.4	Subtropical monsoon climate	22.8	705.6	607.1/189.9
Buenos Aires	-58.4/-34.6	Subtropical monsoon climate	24.7	842.4	730.3/178.7
Sydney	151.2/-33.9	Subtropical monsoon climate	23.2	741.6	639.6/186.4
Santiago de Chile	-70.7/-33.4	Mediterranean climate	22.6	842.4	731.8/168.4
Valparaíso	-71.6/-33	Mediterranean climate	22.3	676.8	581.3/191.8
Perth	115.8/-32	Mediterranean climate	23.6	946.8	826.1/157.6
Cape Town	18.5/-33.9	Mediterranean climate	21.2	867.6	755.6/157.9
San Francisco	-122.5/37.8	Mediterranean climate	10.5	237.6	188.9/204.3
Los Angeles	-118.2/34	Mediterranean climate	15.3	295.2	238.5/216.5
Rome	12.5/41.9	Mediterranean climate	8.7	187.2	144.1/204.3
Athens	23.7/38	Mediterranean climate	11.1	234.0	185.3/207.4

Lisbon	-9.1/38.7	Mediterranean climate	11.5	259.2	208.0/205.3
Beijing	116.4/39.9	Temperate monsoon climate	-3.4	244.8	202.6/149.0
Pyongyang	125.8/39	Temperate monsoon climate	-5.7	223.2	184.0/144.1
Vladivostok	131.9/43.2	Temperate monsoon climate	-11.2	252.0	212.5/121.4
Moscow	37.6/55.8	Temperate continental climate	-5.6	57.6	33.2/169.3
Warsaw	21/52.2	Temperate continental climate	-0.6	68.4	40.8/185.6
New York City	-74/40.8	Temperate continental climate	0.8	205.2	164.6/170.4
Washington D.C.	-77/38.9	Temperate continental climate	2	212.4	170.5/179.0
Ottawa	-75.7/45.4	Temperate continental climate	-9.5	162.0	129.9/140.4
Toronto	-79.4/43.7	Temperate continental climate	-4.6	140.4	108.1/160.4
Montreal	-73.6/45.5	Temperate continental climate	-8.8	144.0	113.2/145.4
London	-0.1/54.5	Temperate marine climate	7.8	75.6	43.0/217.3
Paris	2.4/48.9	Temperate marine climate	6.2	86.4	53.7/209.2
Seattle	-122.3/47.6	Temperate marine climate	6.2	97.2	63.5/207.6
Vancouver	-123/49.2	Temperate marine climate	3.7	111.6	77.9/195.5
Wellington	174.8/-41.3	Temperate marine climate	17.6	666.0	574.5/171.3
Lhasa	91.1/29.7	Plateau mountain climate	0.3	489.6	423.6/125.8

Supplementary Table 4 | Meteorological data and estimated monthly energy saving potential of different cities in July. The critical temperature for determining the working mode of dual-mode device is assumed as 17 °C, approximately equal to the average temperature in Beijing in spring or autumn.

Cities	Longitude (°E)/ Latitude (°N)	Climatic zone	Average temperature (°C)	Global solar radiation (MJ m^{-2})	Energy saving (MJ m^{-2}) (Heating mode/ Cooling mode)
Jakarta	106.8/-6.1	Tropical rainy climate	26.4	543.6	457.2/232.0
Rio de Janeiro	-43.3/-22.9	Tropical savanna climate	20.1	424.8	353.4/219.1
DaresSalaam	39.3/-6.9	Tropical savanna climate	24.1	576.0	488.3/215.7
Mumbai	72.9/19	Tropical monsoon climate	27.6	403.2	328.6/259.1
Kolkata	88.3/22.5	Tropical monsoon climate	29.2	511.2	425.7/251.1
Cairo	31.3/30.1	Tropical desert climate	29.4	792.0	681.0/210.1
Riyadh	46.8/24.7	Tropical desert climate	37.2	817.2	697.8/248.4
Shanghai	121.5/31.2	Subtropical monsoon climate	29.9	532.8	444.8/251.5
Tokyo	139.8/35.7	Subtropical monsoon climate	27	464.4	384.7/246.9
New Orleans	-90.1/30	Subtropical monsoon climate	29	633.6	537.2/231.7
Sao Paulo	-70.7/-33.4	Subtropical monsoon climate	16.9	442.8	371.8/201.6
Buenos Aires	-58.4/-34.6	Subtropical monsoon climate	11.5	284.4	230.9/201.5
Sydney	151.2/-33.9	Subtropical monsoon climate	12.6	288.0	233.6/205.7
Santiago de Chile	-70.7/-33.4	Mediterranean climate	9.4	237.6	189.5/199.7
Valparaíso	-71.6/-33	Mediterranean climate	9.9	205.2	159.8/206.6
Perth	115.8/-32	Mediterranean climate	14.2	338.4	278.5/205.2
Cape Town	18.5/-33.9	Mediterranean climate	13.8	327.6	268.9/205.0
San Francisco	-122.5/37.8	Mediterranean climate	17.3	795.6	692.6/150.5
Los Angeles	-118.2/34	Mediterranean climate	22.3	817.2	709.0/170.7
Rome	12.5/41.9	Mediterranean climate	27.7	781.2	672.5/202.9
Athens	23.7/38	Mediterranean climate	28.3	831.6	717.9/198.4

Lisbon	-9.1/38.7	Mediterranean climate	23	867.6	754.4/166.6
Beijing	116.4/39.9	Temperate monsoon climate	27	532.8	446.9/236.6
Pyongyang	125.8/39	Temperate monsoon climate	24.9	478.8	399.3/234.2
Vladivostok	131.9/43.2	Temperate monsoon climate	18.9	457.2	383.7/208.6
Moscow	37.6/55.8	Temperate continental climate	21.4	615.6	526.2/196.6
Warsaw	21/52.2	Temperate continental climate	21.4	594.0	506.5/199.9
New York City	-74/40.8	Temperate continental climate	26.3	633.6	539.2/218.0
Washington D.C.	-77/38.9	Temperate continental climate	26	655.2	559.0/213.2
Ottawa	-75.7/45.4	Temperate continental climate	22.2	662.4	568.2/193.5
Toronto	-79.4/43.7	Temperate continental climate	22.1	662.4	568.3/193.0
Montreal	-73.6/45.5	Temperate continental climate	21.7	644.4	552.2/193.8
London	-0.1/54.5	Temperate marine climate	20.5	525.6	444.9/205.9
Paris	2.4/48.9	Temperate marine climate	21.3	604.8	516.4/197.8
Seattle	-122.3/47.6	Temperate marine climate	18.8	691.2	596.7/173.1
Vancouver	-123/49.2	Temperate marine climate	18.4	669.6	577.3/174.4
Wellington	174.8/-41.3	Temperate marine climate	9.4	223.2	176.4/201.8
Lhasa	91.1/29.7	Plateau mountain climate	16.6	583.2	499.8/179.2

References

1. Raman, A. P., Anoma, M. A., Zhu, L., Rephaeli, E. & Fan, S. Passive radiative cooling below ambient air temperature under direct sunlight. *Nature* **515**, 540-544 (2014).

2. Zeyghami, M., Goswami, D. Y., Stefanakos, E. A review of clear sky radiative cooling developments and applications in renewable power systems and passive building cooling. *Sol. Energy Mater. Sol. C.* **178**, 115-128 (2018).

3. Zhang, X. et al. Dynamic gating of infrared radiation in a textile. *Science* **363**, 619-623 (2019).

4. Chen, Z., Zhu, L., Li, W. & Fan, S. Simultaneously and Synergistically Harvest Energy from the Sun and Outer Space. *Joule* **3**, 101-110 (2019).

5. Mandal, J. et al. Porous Polymers with Switchable Optical Transmittance for Optical and Thermal Regulation. *Joule* **3**, 3088-3099 (2019).

6. Zhao, H., Sun, Q., Zhou, J., Deng, X. & Cui, J. Switchable Cavitation in Silicone Coatings for Energy-Saving Cooling and Heating. *Adv. Mater.* **32**, 2000870 (2020).

7. Li, X. et al. Integration of daytime radiative cooling and solar heating for year-round energy saving in buildings. *Nat. Commun.* **11**, 6101 (2020).

8. Zhou, L. et al. Hybrid concentrated radiative cooling and solar heating in a single system. *Cell Rep. Phys. Sci.* **2**, 100338 (2021).

9. Luo, H. et al. Outdoor Personal Thermal Management with Simultaneous Electricity Generation. *Nano Lett.* **21**, 3879-3886 (2021).

10. Wu, X. et al. Passive Smart Thermal Control Coatings Incorporating CaF_2/VO_2 Core-Shell Microsphere Structures. *Nano Lett.* **21**, 3908-3914 (2021).

11. Wang, W. et al. Janus Multilayer for Radiative Cooling and Heating in Double-Side Photonic Thermal System. *ACS Appl. Mater. Interfaces* **13**, 42813-42821 (2021).

12. Liu, Y. et al. Automatically Modulated Thermoresponsive Film Based on a Phase-Changing Copolymer. *Chem. Mater.* **33**, 7232-7241 (2021).

13. Wang, S. et al. Scalable thermochromic smart windows with passive radiative

cooling regulation. *Science* **374**, 1501-1504 (2021).

14. Tang, K. et al. Temperature-adaptive radiative coating for all-season household thermal regulation. *Science* **374**, 1504-1509 (2021).
