# Supplementary material for: Temperature-dependent dual-mode thermal management device with net zero energy for year-round energy saving
Source: Nat Commun. 2022 Aug 19;13:4874. doi: 10.1038/s41467-022-32528-1 (PMC9391366; doi:10.1038/s41467-022-32528-1)
Supplement: Supplementary file 3 — Description of Additional Supplementary Files [file 41467_2022_32528_MOESM3_ESM.pdf]

Description of Additional Supplementary Files

File Name: Supplementary Movie 1
Description: Morphological evolution between coiled state and unfolded state of a RC tape-2W SMP laminate.

File Name: Supplementary Movie 2
Description: Switching process between heating mode and cooling mode of a dual-mode device.
